# Primary Renal Carcinoid: Two Rare Cases at a Single Center

**DOI:** 10.7759/cureus.13907

**Published:** 2021-03-15

**Authors:** Emily F Kelly, Zachary M Connelly, Mackenzie J Noonan, Xin Gu, Nazih Khater

**Affiliations:** 1 Urology, Louisiana State University Health Shreveport, Shreveport, USA; 2 Pathology, Louisiana State University Health Shreveport, Shreveport, USA

**Keywords:** primary renal carcinoid, neuroendocrine tumor, carcinoid syndrome

## Abstract

Renal carcinoid tumors are exceedingly rare. These neuroendocrine masses are most frequently found in the gastrointestinal and respiratory tracts. A renal carcinoid tumor has only been documented in around 100 cases. In this article, we report two additional cases in female patients ages 53 and 63. Both tumors were found incidentally on computed tomography scans. Both women underwent radical nephrectomies. Neither has shown evidence of metastasis nor relapse to date; however, the 63-year-old woman was lost to follow-up. In conclusion, upon discovery of the asymptomatic renal mass, renal carcinoid should be a consideration in the differentiation, and if suspected, may be treated with radical nephrectomy as was done in our hospital.

## Introduction

Carcinoid tumors are a rare subset of neuroendocrine tumors with an incidence of 38 for every 1 million persons in the United States [1,2]. Carcinoid tumors are neuroendocrine neoplasms arising most commonly in the gastrointestinal tract and the lungs [3]. Because neuroendocrine cells are not typical of normal renal parenchyma, primary renal carcinoid tumors (PRCTs) are exceedingly rare; however, these tumors remain well-differentiated [4]. Currently, less than 100 such cases have been reported in the entire available literature [5-7]. When these tumors occur, the ability to metastasize is low. In a review of the literature, only 23% were found to metastasize, with lymph node (LN) and liver involvement being the most common locations at 18% each [8].

The paucity of cases, indolent nature of the disease, and illusive findings on imaging complicate the diagnostic process and cause many such cases to go misdiagnosed [9]. Overall, 13% of patients with a renal carcinoid tumor present with carcinoid syndrome [4,10]. This syndrome is a result of the tumor producing excess serotonin. When carcinoid syndrome occurs, patients present with facial flushing, wheezing, blood pressure changes (most commonly hypotension), malnutrition, and diarrhea [10]. The purpose of this report is to add to the overall knowledge base of these tumors.

## Case presentation

Case 1

A 63-year-old female with a past medical history of clinical-stage, IB-grade-2 endometrial adenocarcinoma, status post-total abdominal hysterectomy, and bilateral salpingo-oophorectomy was referred for evaluation of an incidental left renal mass found on surveillance computed tomography of the abdomen and pelvis (CT A/P).

**Figure 1:**
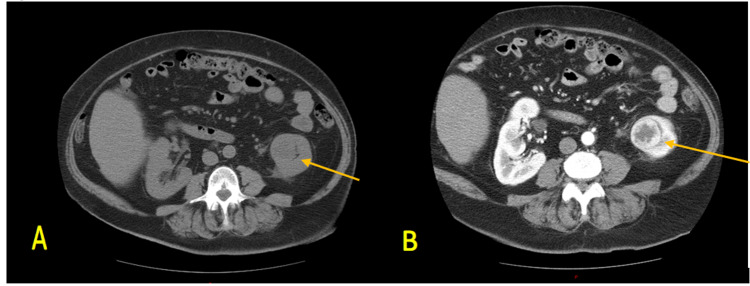
CT scan of the abdomen and pelvis without (A) and with (B) IV contrast with urogram demonstrating a 3.8-cm heterogeneously enhancing lesion of the left lower renal pole as designated by the arrow. CT, computed tomography

On presentation, she denied constitutional symptoms, gross hematuria, flank pain, and lower urinary tract symptoms. The CT urogram scan of the abdomen and pelvis demonstrated a 3.8-cm heterogeneously enhancing lesion of the left lower renal pole (Figure 1). A small number of enlarged retroperitoneal LN was also identified. A two-view (posteroanterior and lateral) chest X-ray (CXR) was also obtained, noting no evidence of metastatic disease. The patient ultimately underwent a left laparoscopic retroperitoneal radical nephrectomy.

**Figure 2:**
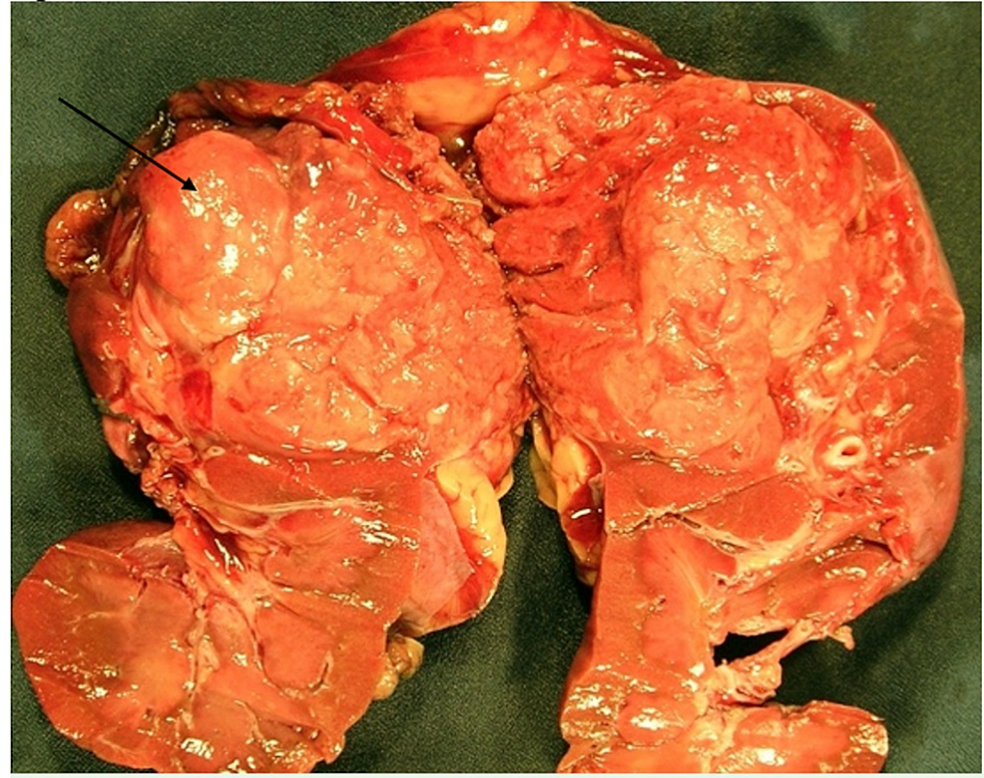
Gross kidney from radical nephrectomy. Gross pathology specimen: 3.5 × 3 × 2-cm yellow-tan mass visualized in the lower pole involving both cortex and medulla as indicated by the arrow. Multiple sections revealed a soft-red, possibly necrotic, lesion in the tumor parenchyma and no renal sinus or vascular involvement.

**Figure 3:**
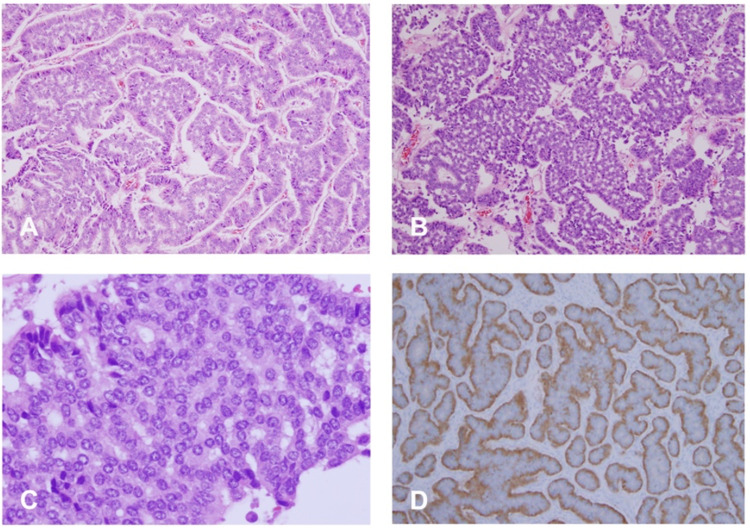
Microscopic evaluation of excised tumor. Variable growth patterns were noted. The tumor cells are arranged as trabeculae, ribbons, or nests (A, B). Solid expansile growth was also noted in focal areas. The tumor cells are uniform with limited cytoplasm and the nuclei are round. Most tumor cells showed “salt and paper” nuclear chromatin (C). Mitotic figures were rare and no geographic necrosis was seen. Immunohistochemistry revealed that the tumor cells were positive for CD56 (3+), synaptophysin (2+), and containing numerous membrane-bound, electron-dense neuroendocrine granules (D).

Gross pathologic examination of the specimen revealed a 3.5 × 3 × 2-cm yellow-tan mass in the lower pole involving both cortex and medulla. No renal sinus or vascular involvement was seen (Figure 2). The initial pathologic diagnosis was clear-cell renal cell carcinoma (RCC), but given the concern for metastatic endometrial cancer to the kidney, we requested that the microscopic examination be reviewed. Repeat examination with immunostaining demonstrated findings typical of a neuroendocrine malignancy with features of carcinoid tumor, arising in the background of clear-cell-type RCC. The tumor cells revealed a positive reaction for CD56 (3+) and synaptophysin (2+). The cells also contained numerous membrane-bound, electron-dense neuroendocrine granules (Figure 3). Microscopically, there was no angiolymphatic invasion, sinus involvement, or extracapsular extension. Final pathologic staging was pT1, N0, Mx with a histopathology grade of G2.

Serial surveillance CT A/P six and 12 months post-operatively demonstrated no evidence of disease with stable retroperitoneal lymphadenopathy. CT A/P at 18 months post-operatively revealed enlarging inter-aortocaval lymphadenopathy, measuring 2.1 × 1.8 cm (compared to 2 × 1.4 cm). Following a discussion with the patient regarding observation with repeat imaging in six months versus biopsy, the patient opted for surveillance.

Over the course of the following year, the LNs continued to enlarge, with the largest measuring up to 3.7 cm. Fine-needle aspiration of an enlarged celiac LN revealed benign hepatocytes and mixed lymphocytes. The patient was followed conservatively with a CT scan every six months for 30 months due to the benign nature of the findings. A positron emission tomography (PET)/CT scan was performed and there was mild uptake noted in the upper abdominal nodes, which was felt to be non-specific; however, low-grade lymphoma could not be excluded as there was no evidence of a metastatic carcinoid tumor. The patient was then lost to follow-up.

Case 2

**Figure 4:**
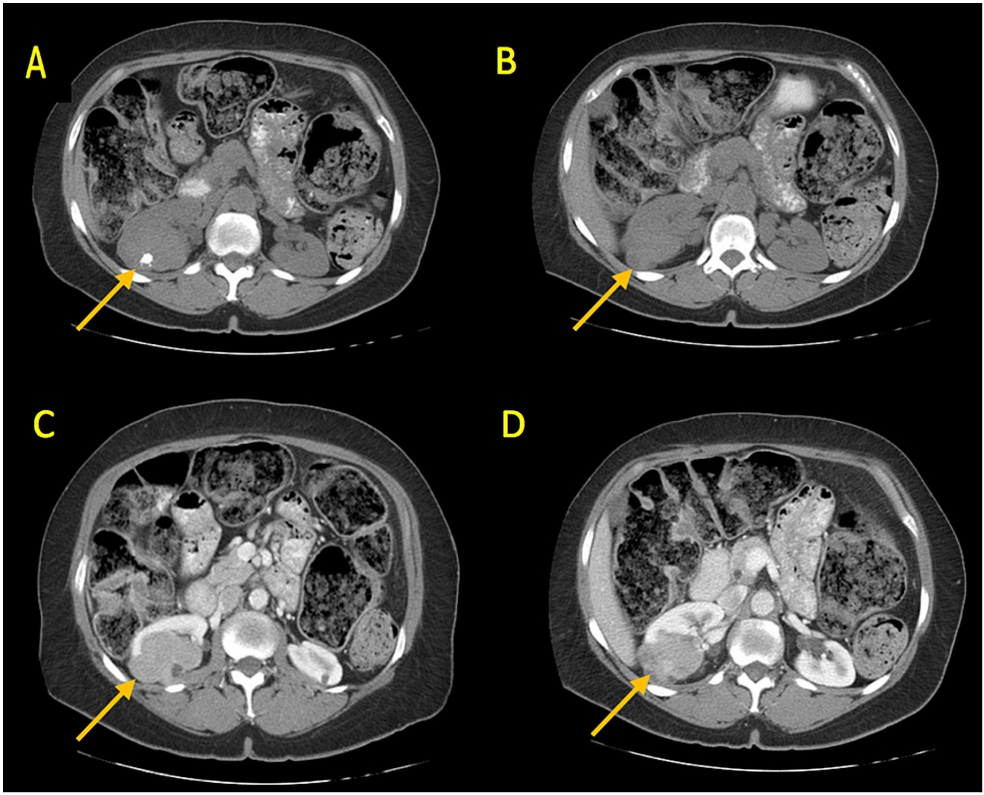
CT scan of abdomen and pelvis. Non-contrast exam demonstrating (A, B) and contrast (C, D) 7-cm prominently solid but enhancing dorsal right mid renal lesion with no evidence of osseous, adrenal, or hepatic metastasis or renal venous invasion or retroperitoneal lymphadenopathy as demonstrated by the arrow.

A 53-year-old female was referred for evaluation of a 7-cm right, intrapolar enhancing renal mass found incidentally on workup for right upper quadrant abdominal pain. Past medical history included hypertension, hyperlipidemia, type II diabetes, and lifelong tobacco use. The patient denied constitutional symptoms, flank pain, gross hematuria, and lower urinary tract symptoms. CT A/P demonstrated a 7-cm prominently solid but enhancing dorsal right mid renal lesion with no evidence of osseous, adrenal, or hepatic metastasis or renal venous invasion or retroperitoneal lymphadenopathy (Figure 4). A CXR was obtained, noting no evidence of metastatic disease. The patient underwent an uncomplicated right hand-assisted laparoscopic nephrectomy.

**Figure 5:**
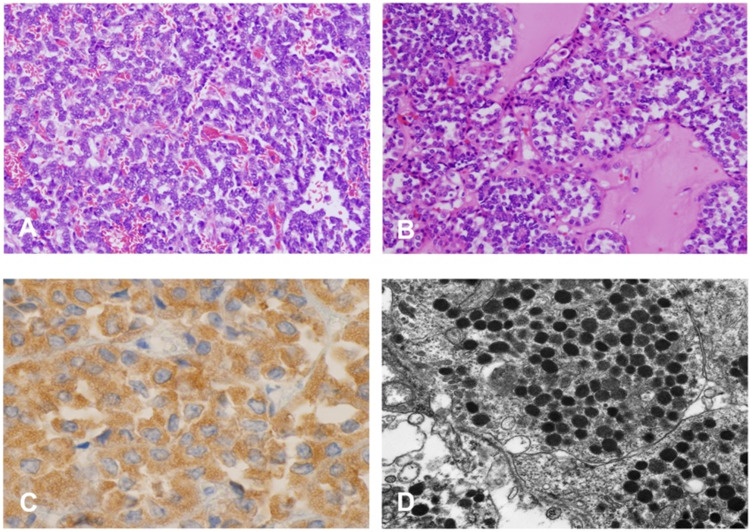
Pathological analysis of the resected tumor. Microscopic evaluation: solid, nest, and sheeting growth of tumor cells were presented in a hyalinized collagenous stroma. The tumor cells were uniform and contain eosinophilic or optically clear cytoplasm with rare mitoses. No angiolymphatic invasion or sinus involvement was identified (A, B). The tumor cells showed a positive reaction for CD56 and synaptophysin in the immunohistochemical study (C) and membrane-bound, electron-dense neuroendocrine granules in ultrastructural examination (D).

Gross pathology revealed a well-demarcated tan solid tumor of the upper pole measuring 6.0 × 6.5 × 3 cm. The tumor appeared to reveal variable parenchyma morphological expression in both growth patterns and cytomorphology. Immunohistochemical stains were positive for CD56 and synaptophysin with membrane-bound, electron-dense neuroendocrine granules consistent with primary renal carcinoid (Figure 5). Final pathologic staging was pT1, Nx, Mx with a histopathology grade of G2.

**Figure 6:**
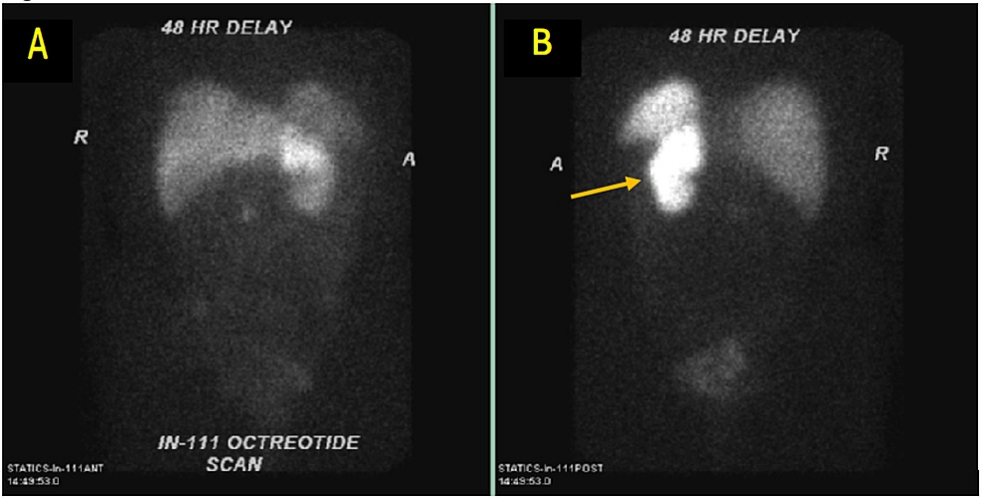
Octreotide uptake scan. Octreotide scan (360-degree view): suspicious focus of abnormal activity in the right abdomen, likely the third portion of the duodenum, which may represent primary carcinoid tumor versus a metastatic lesion. Please note right kidney is surgically absent and the area of the left kidney enhances as octreotide is absorbed normally by native kidney cells.

Four months after the removal of the tumor, serotonin level and urine 5-HIAA were within normal range and a surveillance octreotide scan revealed a suspicious focus of abnormal activity in the right abdomen concerning for primary carcinoid tumor versus metastasis (Figure 6). An upper GI endoscopy revealed a number of gastric ulcers positive for *Helicobacter pylori*. PET scan was unremarkable. CT chest, A/P, performed six months post-operatively showed no evidence of recurrence and resolution of the abdominal mass. We continued with yearly surveillance CT. Serotonin and metabolite levels were obtained twice yearly. The patient remained without recurrence until the last documented follow-up.

## Discussion

PRCTs are very rare neoplasms of neuroendocrine differentiation arising within the renal parenchyma. Patients are often asymptomatic at presentation, but when symptomatic, may have localized or systemic symptoms. Approximately 25-30% are incidental findings, though patients can present with abdominal pain, flank pain, hematuria, constipation, constitutional symptoms, and carcinoid syndromes. These malignancies are more often associated with preexisting renal pathologies, including horseshoe kidney, teratomas, and polycystic kidney disease [9]. There are also reports of these malignancies arising within another primary renal malignancy, including another report of a primary renal carcinoid tumor arising in a background of clear-cell-type RCC [11].

Although radiographic features do not consistently differentiate these neoplasms from other renal masses, CT and octreotide scintigraphy are routinely used to aid in diagnosis and post-operative surveillance of disease [12]. Because of the non-specific clinical and radiologic presentation of these tumors, histology and immunohistology staining is necessary for diagnosis. Uniform collection of ovoid cells forming a trabecular mass with a pseudopapillary structure is the characteristic histologic appearance. Synaptophysin, CgA, S100, CD56, neuron-specific enolase, serotonin, and VIP are common positive markers of neuroendocrine differentiation. Therefore, primary renal carcinoids characteristically stain positive for one or more of the aforementioned markers and negative for urothelial and renal cell markers (CK7, CK20, PAX-2, PAX-8, and CD10) [12-16].

Incidental finding in case 1 and presentation with abdominal pain of another patient in fifth to sixth decade of age is similar to reported cases. Both patients underwent CT A/P, each of which demonstrated a unilateral enhancing renal mass. Subsequent CXRs ruled out metastatic disease in both cases. They both ultimately underwent laparoscopic unilateral radical nephrectomy. Immunohistochemical staining demonstrated positive staining for CD56 and synaptophysin in both cases. Surveillance during the post-operative period differed between patients, despite having the same primary diagnosis and receiving care at the same institution. The non-specific presentation, non-discriminatory pre-operative imaging, surgical treatment, characteristic neuroendocrine pathology, and variable surveillance methods in these cases closely mimic the majority of other rare cases reported in the current literature.

Despite these shared characteristics, some major variability in case presentation still exists and may account for current (and historical) challenges in diagnosis and management. At the time of presentation, case 1 was asymptomatic with an incidental enhancing mass on the lower pole of the left kidney, which was subsequently misdiagnosed as RCC. Upon further pathological workup, a diagnosis of PRCT within the background of clear-cell-type RCC was made. Surveillance included six-month CT A/P for 18 months until the patient was lost to follow-up. Alternatively, case 2 presented with right upper quadrant abdominal pain and an enhancing mass on the upper pole of the right kidney measuring nearly twice the size of the first patient’s mass. However, the course of surveillance for this case was more complex. Surveillance for this patient included a four-month post-operative measurement of serum serotonin and urine 5-HIAA in addition to an octreotide scan with subsequent endoscopy and PET scan. Further testing at six months post-operatively included a CT of the chest and A/P, which was repeated annually thereafter. Measurements of serum markers and urine metabolites were also repeated biannually. Neither patient showed evidence of metastatic disease throughout the surveillance period, although one patient was eventually lost to follow-up. These outcomes are consistent with the well-documented favorable prognosis that follows complete resection of PRCT.

## Conclusions

This case report highlights the variability in presentation leading to difficulty in the diagnosis of renal carcinoid tumors. It is crucial to differentiate this tumor from other renal masses. If a lesion is found within RCC, the management of the patient must be adjusted accordingly. Given the rarity of the condition, there is a lack of follow-up protocols and treatment plans. More research is needed for renal carcinoid tumors.
